# Premenstrual Disorders and Quality of Life in Sweden

**DOI:** 10.1001/jamanetworkopen.2025.33823

**Published:** 2025-09-23

**Authors:** Qiwei Wang, Rebecka Keijser, Yufeng Chen, Hang Yu, Elgeta Hysaj, Sara Hägg, Unnur A. Valdimarsdóttir, Elizabeth Bertone-Johnson, Jurate Aleknaviciute, Donghao Lu

**Affiliations:** 1 Unit of Integrative Epidemiology, Institute of Environmental Medicine, Karolinska Institutet, Stockholm, Sweden; 2 Department of Medical Epidemiology and Biostatistics, Karolinska Institutet, Stockholm, Sweden; 3 Department of Medical Epidemiology and Biostatistics, Karolinska Institutet, Stockholm, Sweden; 4 Center of Public Health Sciences, Faculty of Medicine, University of Iceland, Reykjavík, Iceland; 5 Department of Biostatistics and Epidemiology, School of Public Health and Health Sciences, University of Massachusetts Amherst; 6 Department of Health Promotion and Policy, School of Public Health and Health Sciences, University of Massachusetts Amherst; 7 Department of Psychiatry, Erasmus MC University Medical Center, Rotterdam, the Netherlands

## Abstract

**Importance:**

Premenstrual disorders (PMDs), including premenstrual syndrome (PMS) and premenstrual dysphoric disorder (PMDD), are associated with significant physical and psychological burdens. However, their associations with quality of life (QOL) remain insufficiently studied, particularly with limited consideration of comorbidities and potential confounders.

**Objective:**

To assess the QOL among women with PMDs compared with women without PMDs in Sweden.

**Design, Setting, and Participants:**

This cross-sectional study included participants from the LifeGene cohort, a population-based longitudinal study with linkage to national health care registers. Participants were women aged 15 to 60 years. Data were collected from October 2009 to May 2018 and analyzed from June 2024 to January 2025.

**Exposures:**

PMDs were ascertained using a modified Premenstrual Symptoms Screening Tool, supplemented by diagnoses recorded in Swedish health care registers.

**Main Outcomes and Measures:**

QOL was assessed using the EuroQol 5-Dimensions 3-Levels scale including 5 dimensions: mobility, self-care, usual activities, pain or discomfort, and anxiety or depression. The primary outcome was the total QOL score, with additional analyses excluding the anxiety or depression and pain or discomfort dimensions. Associations between PMDs and total QOL score were evaluated using linear regression models, adjusting for age, socioeconomic status, and potential confounders. Analyses were further stratified by the history of comorbidities. PMS, PMDD, and each QOL dimension were also examined separately.

**Results:**

In this study of 17 284 women, at a mean (SD) age of 32.4 (8.1) years, 1813 women (10.5%) met the criteria for PMDs. Women with PMDs had significantly lower QOL compared with those without PMDs (mean *z* score difference: 0.21; 95% CI, 0.17-0.26). This association persisted in the prospective analysis (mean *z* score difference: 0.34; 95% CI, 0.24-0.45) or after excluding the anxiety or depression and pain or discomfort dimension. PMDD appeared to have a stronger association with reduced QOL than PMS. In the analysis of individual QOL dimensions, PMDs were strongly associated with endorsing anxiety or depression (PR, 1.31; 95% CI, 1.25-1.37) and pain or discomfort (PR, 1.14; 95% CI, 1.08-1.21).

**Conclusions and Relevance:**

In this cross-sectional study of women in Sweden, PMDs, particularly PMDD, were significantly associated with reduced QOL, independent of potential confounders and comorbidities. These findings highlighted the need for both health care and social support to better manage the disease and improve the overall QOL of affected women.

## Key Points

**Question:**

Do women with premenstrual disorders (PMDs) have a lower quality of life compared with women without PMDs?

**Findings:**

In this population-based study of 17 284 women in Sweden, robust evidence from both cross-sectional and prospective analyses found that PMDs were associated with reduced health-related quality of life. This association remained significant after adjusting for potential confounders and stratifying by psychiatric and somatic comorbidities.

**Meaning:**

These findings suggest that PMDs place a substantial burden on quality of life, emphasizing the need for improved disease management to enhance the well-being of affected women.

## Teaser

This cross-sectional study examines the quality of life among women with premenstrual disorders compared with women without premenstrual disorders in Sweden using data from a large population-based longitudinal study.

## Introduction

Premenstrual disorders (PMDs), such as premenstrual syndrome (PMS) and premenstrual dysphoric disorder (PMDD), appear as a blend of affective, behavioral, and physical symptoms during the late luteal phase of the menstrual cycle and mitigate after the onset of menstruation.^1^ PMS affects approximately 20% to 30% of women of reproductive age, while 1.2% to 6.4%^2^ experience PMDD, which is a more severe form characterized by heightened mood disturbances and greater impairment in daily life. Studies have shown that women with PMDD experience impairment comparable with that of women with chronic clinical depression.^3^ Although PMD symptoms are typically confined to the days before menses, the episodic nature of the disorder affects various aspects of life, including social and professional relationships, as well as activities.^4,5^ Furthermore, patients with PMDs are at risk of several negative health consequences,^6^ including both somatic comorbidities (eg, hypertension^6^) and mental outcomes (eg, suicidal behavior^7,8^), leading to significant loss of quality-adjusted life-years.

Previous studies have compared quality of life (QOL) between women with and without PMDs, with sample sizes ranging from 55 to 1008 (eTable 1 in Supplement 1). Data from a clinical trial also suggested that women with PMDD have a lower QOL, even during symptom-free days (ie, in the follicular phase), compared with women without PMDs.^9^ However, most studies were cross-sectional and did not formally assess the association between PMDs and QOL, and the potential contributions of somatic, psychiatric comorbidities, and confounders were not accounted for. To our knowledge, only 2 studies have considered these factors. One prospective study showed that self-reported PMS diagnosis was associated with lowered physical health-related QOL in female nurses.^10^ However, that study focused only on women with a single occupation, which largely limits the generalizability. Another multinational cross-sectional study of 822 participants reported inverse associations of PMDs with both mental and physical health QOL.^11^ However, few somatic comorbidities were accounted for, and heterogeneous results were found between samples (eg, no association between PMDs and mental health QOL in the Austrian sample). To better understand these associations, we conducted a study using a population-based cohort, LifeGene, with linkage to prospectively collected data from registers in Sweden, aiming to assess the QOL among women with PMDs compared with women without PMDs.

## Method

### Data Source

The study used data from LifeGene, a large prospective cohort study in Sweden, which enrolled 39 862 participants, including 24 265 women.^12^ In the LifeGene cohort, individuals aged 18 to 45 years (referred to as index persons) were recruited from the general Swedish population between 2009 and 2019 and were invited to include their household members, such as partners and children. At cohort enrollment, information on demographics, and lifestyle, physical, and psychosocial well-being was collected through a web-based questionnaire, from which we derived data on PMDs, QOL, and covariates.

Subsequently, we sourced data from the National Patient Register (NPR),^13^ the Stockholm Primary Care Register (SPCR),^14^ and the National Prescribed Drug Register (PDR)^15,16^ to identify PMDs diagnosed prior to the participants joining LifeGene. Participants were linked to registers using a unique personal identification number assigned to every resident in Sweden. The NPR provides nationwide data on inpatient visits since 1987 and more than 80% of outpatients since 2001. The SPCR has collected data on patients in public and private primary care services in Stockholm since 2001. Although primary care data are not available for all participants in LifeGene, more than 70% of the participants lived in Stockholm at baseline. The PDR, established in July 2005, contains data on all prescription drugs dispensed in all pharmacies in Sweden. The National Medical Birth Register (MBR) has collected data on virtually all births in Sweden since 1973.^17,18^ Additionally, the Total Population Register (TPR)^19^ was used to obtain information on vital status and demographic characteristics. The Longitudinal Integrated Database for Health Insurance and Labour Market Studies (LISA)^20^ is an annual register that provides socioeconomic data on education, income, and marital status for residents aged 16 years or older. This cross-sectional study was approved by the Swedish Ethical Review Authority. All participants provided informed consent. This study followed the Strengthening the Reporting of Observational Studies in Epidemiology (STROBE) reporting guideline.

### Study Population and Design

We conducted a cross-sectional study followed by a prospective analysis. From the LifeGene cohort, we included 17 793 women aged 15 to 60 years who reported having menstruation in the past year. After excluding 509 individuals with more than 20% of missing data on QOL, a total of 17 284 women were included in the analysis (eFigure in Supplement 1). To address potential reverse causation, a subgroup of the 15 810 study participants with a prospective design was included to assess the association between PMD recorded in registers before LifeGene participation and QOL assessed at enrollment.

### Assessment of PMDs

PMDs were identified either through the LifeGene questionnaire or from diagnoses recorded in health care registers before cohort enrollment. Accordingly, we considered non-PMDs as women with no PMD diagnosis in the registers and reported no significant PMD symptoms in the questionnaire.

At cohort enrollment, PMDs were assessed using a modified version of the Premenstrual Symptom Screening Tool (PSST).^21^ The PSST is a widely used screening tool for PMDs,^22^ aligning with clinical diagnostic criteria, with a positive predictive value of 81%,^23^ and assessing both the severity and impact of premenstrual symptoms.^24^ The questionnaire was modified to begin with 3 screening questions regarding the presence, impact, and cyclical nature of premenstrual symptoms as described in eMethods in Supplement 1. After confirming the screening questions, participants were provided a list of 15 affective and physical symptoms and asked to rate the severity of each symptom from 1 (none) to 4 (severe). An individual’s mean symptom severity was used to impute the missing symptom scores if 20% of symptoms or fewer were missing. The imputation was performed for 233 participants (1.3%). As described elsewhere,^25^ participants were classified as having severe PMS or PMDD based on symptom severity, number of symptoms, and functional impairment (eMethods in Supplement 1).

In addition, clinical or primary care diagnoses of PMDs were retrieved from the NPR and SPCR. To supplement PMD diagnoses made in primary care outside Stockholm, we also identified prescriptions for antidepressants and oral contraceptives indicated for PMD treatment, as previously described.^7^ According to the clinical guidelines, prospective daily symptom ratings for at least 2 consecutive menstrual cycles are required in Sweden.^26^ Diagnoses and treatment codes are provided in eTable 2 in Supplement 1.

### Assessment of QOL

QOL was assessed with the EuroQol 5-Dimensions 3-Levels (EQ-5D-3L) scale at the LifeGene enrollment. The EQ-5D-3L is a standardized instrument for assessing health-related QOL, widely applied in mental health, and with proven validity and reliability.^27,28,29,30^ The first section of the EQ-5D-3L consists of a descriptive system that evaluates health quality at the time of filling the questionnaire across 5 dimensions: mobility, self-care, usual activities, pain or discomfort, and anxiety or depression. Each dimension includes 3 response categories: no problem (score 1), some problem (2), and severe problem (3). If less than 20% of the dimension scores (a maximum of 1 dimension) were missing, the mean score of the remaining dimensions for each individual was used for imputation, applicable to 212 participants (1.2%). To assess overall QOL, we aggregated the scores from the 5 dimensions to obtain a total score (ranging from 5 to 15) as the primary outcome. We also calculate a score without the anxiety and depression or pain and discomfort dimensions because they can be part of PMD symptoms. Additionally, to facilitate comparisons with other studies, we followed the method recommended by EuroQol and converted responses into EQ index scores using the Swedish value set in a complete-case analysis.^31,32^

The second section of the EQ-5D-3L is a modified version of a visual analogue scale (VAS). This scale captures the respondent’s self-rated overall health status on a vertical visual analogue scale ranging from 0 to 10, where 0 represents the “worst health you can imagine” and 10 represents the “best health you can imagine.”

### Covariates

Demographic variables, including age and region of residence, were retrieved from the TPR, and country of birth was retrieved from both the TPR and LifeGene. Individualized household disposable income at cohort enrollment was obtained from LISA, while civil status and educational attainment were sourced from LISA and supplemented by LifeGene. Potential confounders, including height and weight (used to calculate body mass index [BMI]), smoking status, alcohol intake, childhood abuse, and stressful life events were assessed at the time of cohort enrollment through questionnaires (eMethods in Supplement 1). Data on parity was derived by combining information from the MBR and the TPR.

PMDs are often comorbid with psychiatric disorders,^33^ which have been associated with lower QOL.^34^ Depending on whether they occur before or after PMDs, psychiatric disorders might confound or mediate the association between PMDs and QOL. Furthermore, varying chronic diseases, such as diabetes and cardiovascular disease, have a significant impact on QOL.^35,36^ We used the Charlson Comorbidity Index (CCI) to assess somatic comorbidities.^37^ Only psychiatric and somatic conditions diagnosed before their enrollment and recorded in NPR were included (codes in eTable 2 in Supplement 1).

### Statistical Analysis

At cohort enrollment, characteristics of women with and without PMDs were summarized using descriptive statistics. Linear regression models were applied to assess the association of PMDs with the overall QOL score and the score excluding the anxiety and depression or pain and discomfort dimensions. To address potential reverse causation, we conducted a prospective analysis restricting PMDs to those diagnosed before cohort enrollment. Considering potential confounders, 3 models were constructed: model 1 was a crude model; model 2 adjusted for demographic and socioeconomic factors; and model 3 (primary model) additionally adjusted for BMI, smoking status, alcohol intake, childhood abuse, adulthood stressful life events, and parity.

In addition, we estimated prevalence ratios (PRs) of each QOL dimension (coded as “yes” if any endorsement) using the robust Poisson regression model.^38^ As PMDD and PMS may have varying impacts on QOL, we conducted analyses by PMDD and PMS separately. Moreover, we conducted stratified analyses based on psychiatric or somatic comorbidities to illustrate potential risk modification.

To further evaluate the robustness of our primary findings, we used the VAS score as a supplementary measure of overall QOL. Additionally, we restricted the analysis to participants whose PMDs were confirmed by both clinical diagnoses and questionnaire assessments, presumably with a higher validity for PMD ascertainment. Finally, we performed a complete case analysis on participants who completed every item on the QOL questionnaire, using the EQ-value, and total score as outcome measures.

The data were prepared using R software (version 4.4.0), and statistical analyses were performed using Stata. *P* < .05 was considered statistically significant. Data were collected from October 2009 to May 2018 and analyzed from June 2024 to January 2025.

## Results

### Characteristics

In this study of 17 284 women, at a mean (SD) age of 32.4 (8.1) years, 1813 women (10.5%) met the criteria for PMDs. Compared with women without PMDs, those with PMDs were somewhat older and more likely to be overweight (but not obese) and less likely to be underweight, born abroad, and living in Stockholm and to have lower income yet higher education attainment (Table 1). They were also more likely to smoke, report experiences of childhood abuse and adulthood stressful life events, and have given birth as well as to have psychiatric and somatic comorbidities. The characteristics of the excluded women due to missing more than 20% of the QOL questionnaire items were similar to those included (eTable 3 in Supplement 1).

**Table 1.** Characteristics of Participants With and Without Premenstrual Disorders

Characteristics at enrollment	Participants, No. (%)	
	PMDs (n = 1813)	No PMDs (n = 15 471)
Age, mean (SD), y	34.0 (8.1)	32.2 (8.5)
15-24	3136 (20.3)	223 (12.3)
25-34	6702 (43.3)	786 (43.4)
35-44	3971 (25.7)	584 (32.2)
45-54	1608 (10.4)	213 (11.8)
55-60	54 (0.4)	7 (0.4)
BMI		
<18.5	32 (1.8)	520 (3.4)
18.5-25	1286 (70.9)	10 828 (70.0)
25-29	299 (16.5)	2282 (14.8)
≥30	53 (2.9)	465 (3.0)
Unknown	143 (7.9)	1376 (8.9)
Country of birth		
Sweden	1571 (86.6)	13 865 (89.6)
Other	243 (13.4)	1606 (10.4)
Region of residence		
Stockholm	1461 (80.6)	11 824 (76.4)
Other	352 (19.4)	3647 (23.6)
Civil status		
Partnered	680 (37.5)	5939 (38.4)
Single	1133 (62.5)	9527 (61.6)
Unknown	0	5 (0.03)
Education level		
Presecondary	15 (0.8)	141 (0.9)
Secondary	349 (19.3)	2984 (19.3)
Postsecondary	1253 (69.1)	11 005 (71.1)
Postgraduate	196 (10.8)	1335 (8.6)
Unknown	0	6 (0.04)
Income^a^		
Q1	475 (26.2)	3837 (24.8)
Q2-Q3	929 (51.24)	7698 (49.8)
Q4	408 (22.5)	3900 (25.2)
Unknown	1 (0.1)	36 (0.2)
Alcohol intake		
Never	57 (3.1)	512 (3.3)
Monthly	933 (51.5)	8096 (52.3)
Weekly	790 (43.6)	6458 (41.7)
Unknown	33 (1.8)	405 (2.6)
Smoking status		
Never	467 (25.8)	5371 (34.7)
Former smokers	1149 (63.4)	8694 (56.2)
Current smokers	184 (10.2)	1233 (8.0)
Unknown	13 (0.7)	173 (1.1)
Childhood abuse		
Yes	476 (26.3)	2507 (16.2)
No	1325 (73.1)	12 524 (81.0)
Unknown	12 (0.7)	440 (2.8)
Stressful life events, No.		
0	217 (12.0)	3106 (20.1)
1	360 (19.9)	3913 (25.3)
2-3	654 (36.1)	5135 (33.2)
>3	567 (31.3)	2866 (18.5)
Unknown	15 (0.8)	451 (2.9)
Parity		
0	1066 (58.8)	10 155 (65.6)
1-2	599 (33.0)	4369 (28.2)
≥3	148 (8.2)	947 (6.1)
Psychiatric comorbidities		
Yes	599 (33.0)	3375 (21.8)
No	1214 (67.0)	12 096 (78.2)
Somatic comorbidities^a^		
Yes	256 (14.12)	1680 (10.86)
No	1557 (85.88)	13 791 (89.14)

Abbreviations: BMI, body mass index (calculated as weight in kilograms divided by height in meters squared); PMD, premenstrual disorder; Q, quartile.

^a^ Somatic comorbidities were assessed by the Charlson Comorbidity Index score.

### PMDs and Quality of Life

Compared with women without PMDs, women with PMDs had a lower QOL when assessing the total score (higher score indicates lower QOL; fully-adjusted difference in mean *z* score: 0.21; 95% CI, 0.17-0.26) (Table 2). Attenuated yet significant associations were observed after excluding the anxiety and depression item from the total score (mean score difference, 0.10; 95% CI, 0.05-0.15), whereas excluding the pain and discomfort item yielded materially unchanged results (mean score difference, 0.21; 95% CI, 0.16-0.26). In the prospective analysis restricting PMDs to 339 women diagnosed before enrollment or QOL assessment (mean [SD] interval, 3.64 [2.98] years), associations were comparable and even somewhat stronger (Table 2). When assessing the 5 QOL dimensions individually, we found significant associations of PMDs with endorsing anxiety and depression (fully adjusted PR, 1.31; 95% CI, 1.25-1.37) and pain and discomfort (fully adjusted PR, 1.14; 95% CI, 1.08-1.21) but no associations with mobility, self-care, and usual activity (Table 3).

**Table 2.** Association Between PMDs and QOL

Design	Mean (SD)		Difference in mean score (95% CI)		
	No PMDs	PMDs	Model 1^a^	Model 2^b^	Model 3^c^
**Cross-sectional design**					
No.	15 471	1813	NA	NA	NA
Total score^d^	−0.03 (0.99)	0.27 (1.03)	0.30 (0.25 to 0.35)^e^	0.28 (0.23 to 0.33)^e^	0.21 (0.17 to 0.26)^e^
Total score without anxiety and depression item	−0.02 (0.99)	0.16 (1.08)	0.18 (0.13 to 0.23)^e^	0.15 (0.10 to 0.20)^e^	0.10 (0.05 to 0.15)^e^
Total score without pain and discomfort item	−0.03 (0.99)	0.24 (1.04)	0.27 (0.23 to 0.32)^e^	0.27 (0.22 to 0.32)^e^	0.21 (0.16 to 0.26)^e^
**Prospective design** ^f^					
No.	15 471	339	NA	NA	NA
Total score	−0.03 (0.99)	0.37 (1.01)	0.40 (0.30 to 0.51)^e^	0.38 (0.28 to 0.49)^e^	0.34 (0.24 to 0.45)^e^
Total score without anxiety and depression item	−0.02 (0.99)	0.19 (1.08)	0.21 (0.10 to 0.31)^e^	0.14 (0.04 to 0.25)^e^	0.12 (0.01 to 0.22)^e^
Total score without pain and discomfort item	−0.03 (0.99)	0.39 (1.04)	0.42 (0.31 to 0.52)^e^	0.44 (0.34 to 0.55)^e^	0.40 (0.30 to 0.51)^e^

Abbreviations: EQ-5D-3L, EuroQol 5-Dimensions 3-Levels; NA, not applicable; PMD, premenstrual disorder; QOL, quality of life.

^a^ Model 1 was a crude model.

^b^ Model 2 was adjusted for age, country of birth, region of residence, civil status, educational level, and income.

^c^ Model 3 was further adjusted for body mass index, alcohol intake, smoking status, childhood abuse, stressful life events, and parity.

^d^ The total score was calculated by summing responses to all 5 questions in the EQ-5D-3L scale, with a higher score indicating poorer quality of life, and then converted to a *z* score.

^e^ *P* < .05.

^f^ The prospective design was restricted to PMDs diagnosed before QOL measurement, with a mean (SD) interval of 3.64 (2.98) years.

**Table 3.** Association Between PMDs and Individual Item of Quality of Life^a^

Individual item^b^	With problem, No. (%)		PR (95% CI)		
	No PMDs, n = 15 471	PMDs, n = 1813	Model 1^c^	Model 2^d^	Model 3^e^
Mobility	263 (1.7)	41 (2.3)	1.33 (0.96-1.84)	1.21 (0.88-1.68)	1.09 (0.79-1.51)
Self-care	43 (0.3)	6 (0.3)	1.19 (0.51-2.79)	1.10 (0.47-2.56)	1.12 (0.47-2.66)
Usual activities	520 (3.4)	76 (4.2)	1.25 (0.99-1.58)	1.20 (0.95-1.52)	1.02 (0.80-1.29)
Pain or discomfort	5469 (35.4)	812 (44.8)	1.27 (1.20-1.34)^f^	1.22 (1.15-1.29)^f^	1.14 (1.08-1.21)^f^
Anxiety or depression	6263 (40.5)	1019 (56.2)	1.39 (1.33-1.45)^f^	1.39 (1.33-1.46)^f^	1.31(1.25-1.37)^f^

Abbreviations: EQ-5D-3L, EuroQol 5-Dimensions 3-Levels; PMD, premenstrual disorder; PR, prevalence ratio.

^a^ In this analysis, a modified Poisson regression with robust standard errors was applied to estimate the prevalence ratio.

^b^ The EQ-5D-3L scale includes 5 individual items, with each dimension having 3 response categories: no problem (1), some problem (2), and severe problem (3). Due to data imputation resulting in noninteger values, we used 2 as the cutoff to define with problem and 2 or more to define without problem.

^c^ Model 1 was a crude model.

^d^ Model 2 was adjusted for age, country of birth, region of residence, civil status, educational level, and income.

^e^ Model 3 was further adjusted for body mass index, alcohol use, smoking status, childhood abuse, stressful life events, and parity.

^f^ *P* < .05.

When analyzing the total score according to PMD subtypes, both PMS and PMDD were associated with lower QOL, with a stronger association observed for PMDD (PMS: mean score difference, 0.16; 95% CI, 0.04-0.28; PMDD: mean score difference; 0.22; 95% CI, 0.16-0.27) (Table 4). After excluding the anxiety and depression item, the associations were attenuated and remained significant for PMDD (mean score difference, 0.10; 95% CI, 0.05-0.15) but not for PMS. Excluding the pain and discomfort item yielded comparable associations for both PMS (mean score difference, 0.13; 95% CI, 0.01-0.26) and PMDD (mean score difference, 0.21; 95% CI, 0.16-0.26).

**Table 4.** Association of PMD Subtypes With QOL^a^

PMD subtypes	Participants, No. (%), N = 17 239	QOL scores					
		Total		Total without anxiety or depression item		Total without pain or discomfort item	
		Mean (SD)	Mean score difference (95% CI)^b^	Mean (SD)	Mean score difference (95% CI)^b^	Mean (SD)	Mean score difference (95% CI)^b^
No PMDs	15 471 (88.5)	−0.03 (0.99)	1 [Reference]	−0.02 (0.99)	1 [Reference]	−0.03 (0.99)	1 [Reference]
PMS	241 (1.4)	0.18 (1.00)	0.16 (0.04 to 0.28)^c^	0.16 (1.05)	0.10 (−0.02 to 0.23)^c^	0.12 (1.04)	0.13 (0.01 to 0.26)^c^
PMDD	1527 (8.6)	0.28 (1.03)	0.22 (0.16 to 0.27)^c^	0.16 (1.07)	0.10 (0.05 to 0.15)^c^	0.25 (1.03)	0.21 (0.16 to 0.26)^c^

Abbreviations: PMD, premenstrual disorder; PMDD, premenstrual dysphoric disorder; PMS, premenstrual syndrome; QOL, quality of life.

^a^ In this analysis, PMDs identified via the questionnaire were classified as PMDD or PMS regardless of clinical diagnosis in registers. Those classified as non-PMD by the questionnaire, which only screened for PMDD and severe PMS, but had a clinical diagnosis before cohort enrollment were classified as PMS. If the questionnaire data were missing (45 [0.3%]), they were not included in this analysis.

^b^ Models were adjusted for age, country of birth, region of residence, civil status, educational level, income, body mass index, alcohol drinking, smoking status, childhood abuse, stressful life events, and parity.

^c^ *P* < .05.

### Additional Analyses

In the stratified analysis, individuals with both PMDs and a history of psychiatric or somatic comorbidities had a lower QOL compared with those without either condition when assessing the total score (Table 5). However, no statistically significant interactions were observed between PMDs and psychiatric or somatic comorbidities. Comparable results were found when excluding anxiety and depression or pain and discomfort from the total score (eTable 7 in Supplement 1). Notably, significant interaction was found between PMDs and somatic comorbidities when the anxiety and depression item was removed from the total score (*P* for interaction = .01).

**Table 5.** Association Between PMDs and Quality of Life Stratified by Comorbidities

Comorbidities	Total score		*P* value for interaction^b^
	Mean, SD	Difference in mean score (95% CI)^a^	
**Psychiatric comorbidities**			
No			
No PMDs	−0.16 (0.89)	1 [Reference]	NA
PMDs	0.09 (0.93)	0.19 (0.13 to 0.24)^c^	
Yes			
No PMDs	0.44 (1.17)	0.49 (0.45 to 0.52)^c^	.41
PMDs	0.63 (1.13)	0.63 (0.55 to 0.71)^c^	
**Somatic comorbidities**			
No			
No PMDs	−0.07 (0.96)	1 [Reference]	NA
PMDs	0.21 (0.97)	0.19 (0.14 to 0.24)^c^	
Yes			
No PMDs	0.26 (1.18)	0.24 (0.19 to 0.29)^c^	.18
PMDs	0.60 (1.28)	0.52 (0.40 to 0.64)^c^	

Abbreviations: NA, not applicable; PMD, premenstrual disorder.

^a^ Models were adjusted for age, country of birth, region of residence, civil status, educational level, income, body mass index, alcohol drinking, smoking status, childhood abuse, stressful life events, and parity.

^b^ *P* values for interaction where obtained from F-tests of linear hypotheses in the Ordinary Least Squares regression model.

^c^ *P* < .05.

Using the VAS score as an alternative assessment for QOL, we also found that women with PMD had a lower QOL (eTable 4 in Supplement 1). In a sensitivity analysis restricting PMDs to those confirmed by both clinical diagnoses and questionnaire assessments, we obtained comparable results to the primary analysis (eTable 5 in Supplement 1). Finally, in the complete-case analysis limited to women who had complete QOL data, the results of the total score were consistent with the primary analysis (eTable 6 in Supplement 1). When applying EQ value as an alternative estimate, an inverse association between PMD and QOL was noted (eTable 6 in Supplement 1).

## Discussion

In this population-based study of 17 284 women in Sweden, we found robust evidence from both cross-sectional and prospective analyses that PMDs are associated with reduced health-related QOL. This association remained significant after adjusting for a range of potential confounders and stratifying by psychiatric and somatic comorbidities. The association between PMDs and QOL was particularly notable in the dimension of anxiety and depression followed by pain and discomfort. A more pronounced association was suggested for PMDD, which was largely accounted for by the anxiety and depression dimension.

To our knowledge, this study is the largest population-based study illustrating an association between PMDs and a lower health-related QOL. Our findings align with 2 previous studies. After adjusting for age and other chronic diseases, Wei et al^10^ reported an association between self-reported diagnosis of PMS and lower physical-related QOL (main score difference, −0.41; standard error, 0.04). However, this study did not investigate psychological aspects of QOL or PMDD. Heinemann et al^11^ found that PMDD and moderate-to-severe PMS were associated with lower psychological (adjusted OR across countries: 0.47; 95% CI, 0.35-0.63) and physical (0.47; 95% CI, 0.40-0.73) QOL. However, both studies did not account for potential confounding from childhood abuse and stressful life events, which have been strongly associated with both PMDs and QOL.^38,39^ Therefore, the present study provides evidence with a more comprehensive adjustment for potential confounders and stratification by somatic and psychiatric comorbidities. In addition, we adopted a prospective study design to address the potential reverse causation inherent in cross-sectional studies.

The association found in this study between PMDs and QOL (mean *z* score difference, 0.21) was higher than the impact of neuroticism (mean *z* score difference, 0.16),^40^ yet lower than alcohol use disorder (mean *z* score difference, 0.34).^41^ Considering that some questionnaires on QOL were completed during the PMD symptom-free period, the observed association may in fact be underestimated. Given the relatively high prevalence of PMD and its cyclic nature, its impact on QOL remains a significant public health concern.

PMD-associated reduction of QOL was most pronounced in the dimensions of anxiety and depression, consistent with their role as core symptoms of PMDs. Moreover, depression and anxiety were the most prevalent psychiatric comorbidities among patients with PMDs.^33,42^ Indeed, we observed an attenuated yet significant association between PMDs and the QOL score after removing the anxiety and depression dimension. Previous studies have often excluded women with a history of other psychiatric disorders from PMD research,^43,44,45,46^ resulting in limited evidence available for this group. In this study, the stratified analysis showed that women with both PMDs and a history of psychiatric comorbidities tended to have markedly lower QOL. Although no significant interaction was observed, the findings underscore the importance of improving the QOL in this vulnerable group and the need for targeted preventive strategies.

In this study, pain and discomfort were also found to be significant contributors to the reduction in QOL among individuals with PMDs. These symptoms can arise not only from PMD-related characteristics, such as premenstrual bloating, limb swelling, and breast tenderness, but also from somatic comorbidities. Indeed, a more pronounced association was indicated among individuals with both PMDs and somatic comorbidities. For instance, hypertension is more common among women with PMDs.^47^ Dysfunction of the renin-angiotensin-aldosterone system (RAAS) contributes to hypertension by altering sodium balance, blood volume, and arterial constriction regulation.^48^ Additionally, RAAS dysfunction appears to be associated with premenstrual symptoms, such as bloating and swelling, which are key contributors to bodily pain and indirectly affect QOL.^49^

Both PMS and PMDD are associated with a lower QOL, consistent with existing literature.^11^ However, this study advances current knowledge by directly comparing the differential impacts of PMS and PMDD on QOL, suggesting a stronger association for PMDD. Specifically, this stronger association appears to be primarily driven by symptoms of anxiety and depression. In other words, more severe depressive symptoms, irritability, and tension before menstruation of PMDD, as required by the diagnostic criteria, may lead to a negative impact on QOL. It is worth noting that the observed prevalence of PMDD in our study was approximately 9.0%, which is higher than estimates based on prospective assessments (pooled prevalence, 1.3%) yet comparable with those based on retrospective (single-time point) symptom reports (pooled prevalence, 7.7%).^50^ The use of the PSST may overestimate prevalence compared with prospective daily ratings.^51^

### Study Limitations

This study has limitations. First, the PSST assessment cannot confirm the diagnosis of PMDs, which requires prospective daily ratings over a minimum of 2 symptomatic cycles.^52^ Although prospective symptoms charting is required by clinical guidelines in Sweden, we lacked such information in registers. To alleviate such concern, we conducted a sensitivity analysis using PMDs confirmed by both self-report and clinical diagnoses, and comparable results were yielded. Second, the EQ-5D-3L scale only evaluates physical and mental aspects of QOL, lacking a comprehensive assessment of the broader social and environmental dimensions. In the meantime, PMD symptoms inherently overlap with certain QOL dimensions, particularly anxiety or depression and pain or discomfort. While leave-one-out analysis mitigated this issue, some degree of measurement overlap may remain. However, we have yielded similar or even stronger associations in the prospective analysis with a mean (SD) interval of 3.64 (2.98) years between PMD diagnosis and QOL assessment. Given that the PMD symptoms and severity can vary across cycles,^53^ this finding supports that the association was not completely driven by the overlapping measurement. Third, compared with the general Swedish population, LifeGene participants were slightly younger, had higher educational attainment, and were more likely to live alone.^12^ However, the prevalence of PMDs, mostly PMDD and severe PMS, is consistent with the estimates from the general population.^9^

## Conclusions

In this study, PMDs, particularly PMDD, were significantly associated with reduced QOL, with the highest prevalence observed in the dimensions of anxiety and depression and pain and discomfort. While future research using prospective data are warranted to further clarify these associations, our findings underscore the substantial burden of PMDs and highlight the need to further improve the disease management and enhance women’s well-being.

## Notes

Supplement 1. **eTable 1. **Literature review on premenstrual disorder and quality of life **eTable 2. **Codes for identification of premenstrual disorders, psychiatric, and somatic comorbidities
eMethods.
**eTable 3. **Comparison of characteristics between included and excluded women **eTable 4. **Association between PMDs and quality of life, assessed by VAS score **eTable 5.** Association between PMDs and quality of life, restricted to PMDs ascertained in both clinical diagnoses and questionnaire assessment **eTable 6.** Association between PMDs and quality of life, a complete case analysis **eTable 7.** Sensitivity analysis: association between PMDs and quality of life stratified by comorbidities

Supplement 2.
Data Sharing Statement
