# Supplementary material for: Premenstrual Disorders and Quality of Life in Sweden
Source: JAMA Netw Open. 2025 Sep 23;8(9):e2533823. doi: 10.1001/jamanetworkopen.2025.33823 (PMC12457971; doi:10.1001/jamanetworkopen.2025.33823)
Supplement: Supplement 1. — eTable 1. Literature review on premenstrual disorder and quality of life eTable 2. Codes for identification of premenstrual disorders, psychiatric, and somatic comorbidities eMethods. eTable 3. Comparison of characteristics between included and excluded women eTable 4. Association between PMDs and quality of life, assessed by VAS score eTable 5. Association between PMDs and quality of life, restricted to PMDs ascertained in both clinical diagnoses and questionnaire assessment eTable 6. Association between PMDs and quality of life, a complete case analysis eTable 7. Sensitivity analysis: association between PMDs and quality of life stratified by comorbidities [file jamanetwopen-e2533823-s001.pdf]

Supplemental Online Content

Wang Q, Keijser R, Chen Y, et al. Premenstrual disorders and quality of life in Sweded. *JAMA Netw Open*. 2025;8(9):e2533823.
doi:10.1001/jamanetworkopen.2025.33823

eTable 1. Literature review on premenstrual disorder and quality of life

eTable 2. Codes for identification of premenstrual disorders, psychiatric, and somatic comorbidities

eMethods.

eTable 3. Comparison of characteristics between included and excluded women

eTable 4. Association between PMDs and quality of life, assessed by VAS score

eTable 5. Association between PMDs and quality of life, restricted to PMDs ascertained in both clinical diagnoses and questionnaire assessment

eTable 6. Association between PMDs and quality of life, a complete case analysis

eTable 7. Sensitivity analysis: association between PMDs and quality of life stratified by comorbidities

This supplemental material has been provided by the authors to give readers additional information about their work.

© 2025 Wang Q et al. *JAMA Network Open*.

eTable 1 Literature review on premenstrual disorder and quality of life

First author last name, year	Study design<br>Data source<br>Study population	Exposure	Outcome	Main findings	Notes
Bhuvaneswari, 2019	**Study Design:** Cross-sectional descriptive study<br>**Data Source:** Self-administered questionnaires<br>**Study Population:**<br>*Description:* Women college students from a single college in Puducherry<br>*Size:* 300 students aged 18–22 years	- Premenstrual Syndrome (PMS): Evaluated using the Shortened Premenstrual Assessment Form (SPAF), with a score >27 defining PMS;<br>- Premenstrual Dysphoric Disorder (PMDD): Evaluated using a checklist based on DSM-5 criteria.	Quality of Life (QoL): Evaluated using the SF-36 Health Survey, which assesses physical, mental, emotional, and social domains.	Prevalence: 62.7% (PMS) and 65.7% (PMDD) prevalence among participants.<br>- Common symptoms included body, muscle, and joint aches, followed by abdominal discomfort, with about half experiencing affective symptoms like irritability, anxiety, and mood swings.<br>- Mean (SD) general health scores were 71.7 (23.89) for participants without PMS and 27.9 (17.18) for those with PMS.<br>- The SPAF score correlated strongly with the PMDD checklist item count (r = 0.748).	Confounders not adjusted in PMS/PMDD-QoL association
Farrokh-	**Study Design:** Cross-	PMS: Evaluated using	QoL: Evaluated using	- Prevalence of PMS: 39.4%	Confounders

© 2025 Wang Q et al. *JAMA Network Open*.

First author last name, year	Study design<br>Data source<br>Study population	Exposure	Outcome	Main findings	Notes
Eslamlou, 2015	sectional study<br>**Data Source:** Self-administered questionnaires<br>**Study Population:**<br>*Description: 142* Unmarried Female medical students at Urmia University of Medical Sciences, Iran	DSM-IV criteria, and symptom severity assessed via Premenstrual Syndrome Scale (PMSS, completed over two prospective menstrual cycles).	the Persian version of the World Health Organization's Quality of Life (WHOQOL-BREF) across physical, psychological, social, and environmental domains.	- Significant decrease in mental health (p = 0.02) and environmental health (p = 0.002) domains with increasing PMS severity.<br>- No significant relationship between PMS and physical or social QoL domains.	not adjusted in PMS-QoL association
Pinar, 2010	**Study Design**: Descriptive, cross-sectional study<br>**Data Source**: Questionnaire-based survey<br>**Study Population**:<br>Female college students aged 18–28 (n = 316)<br>Participants were from the Medical Sciences Faculty of Baskent University.	PMS: Assessed using PMSS, based on DSM-III and DSM-IV criteria.	QoL: evaluated by WHOQOL-BRIEF-TR	- PMS was detected in 72.1% of students.<br>- PMS was significantly higher in those with menstrual irregularity, dysmenorrhea, high caffeine intake, smoking, and alcohol use.<br>- Students with PMS had significantly lower overall life quality (F = 82.583, p = 0.000), especially in physical health (F = 21.286, p < 0.001) and environmental domains (F = 87.947, p < 0.001).	Confounders not adjusted in PMS-QoL association
Goker, 2015	**Study Design**: Cross-sectional study<br>**Data Source**: Questionnaire-based survey<br>**Study Population**:	PMS: Evaluated using Symptom Calendar (two consecutive menstrual cycles) based on ACOG	QoL: Evaluated using SF-36	- Most frequent symptoms: Abdominal bloating (89.5%), irritability (88.3%), and breast tenderness (82.6%).<br>- Stress, alcohol consumption, fat-	Covariate Adjustment: Family history of PMS

© 2025 Wang Q et al. *JAMA Network Open*.

First author last name, year	Study design<br>Data source<br>Study population	Exposure	Outcome	Main findings	Notes
	**Description**: Female medical students at Celal Bayar University, Turkey<br>**Size**: 172 students who completed the survey	criteria		rich diets, and family history significantly increased PMS severity and impacted QoL.<br>- Severe PMS was associated with significantly lower scores in general health, emotional functioning, and social functioning.<br>- Family history and younger age of menarche (<13 years) were significant risk factors for PMS severity.	
Victor, 2019	**Study Design:** Cross-sectional study<br>**Data Source:** self-reported data from Faculdade Pernambucana de Saúde, Recife, Brazil<br>**Study Population**: 642 university students aged 18 years or older with regular menses, from health-related courses in Recife, Brazil.	PMS and PMDD:<br>PMS: Diagnosis based on American College of Obstetricians and Gynecologists (ACOG) criteria;<br>PMDD: DSM-5 criteria was used for PMDD diagnosis.	QoL: the WHOQOL-BREF questionnaire (the validated version in Brazil)	- 49.9% of students experienced PMS, with 26.6% meeting criteria for PMDD.<br>- Quality of life scores were significantly lower in the physical and mental domains for students with mild PMS or PMDD compared to those without PMS.<br>- Differences were also significant in the social relationships and environmental domains for students with mild PMS.	Confounders not adjusted in PMS-QoL association
İşik, 2016	**Study Design**: Cross-sectional study<br>**Data Source**: self-reported	PMS: PMS scale revised from the Diagnostic and	QoL: Life Quality Test-Short Form (SF 36).	- 84.5% of students were diagnosed with PMS.<br>- Physical functioning, physical	Confounders not adjusted in PMS-QoL

© 2025 Wang Q et al. *JAMA Network Open*.

First author last name, year	Study design<br>Data source<br>Study population	Exposure	Outcome	Main findings	Notes
	data from Health campus of a state university in Turkey<br>**Study Population (Description and Size)**: 608 female health science students aged 17-36 years, excluding those with amenorrhea, chronic pelvic pain, or on oral contraceptives.	Statistical Manual of Mental Disorders (DSM - III);		role, general health, social functioning, mental health, and vitality scores decreased significantly with increasing PMS severity (p < 0.001).<br>- Bodily pain scores increased significantly as PMS severity increased.<br>- No significant differences were found among PMS groups in the emotional role domain (p = 0.104).	association
Mahmood, 2023	**Study Design:**<br>Descriptive study<br>**Data Source:**<br>Survey data from Fatima Jinnah Medical University, Lahore, Pakistan<br>**Study Population:**<br>635 female medical students from 3rd to final year MBBS<br>Inclusion criteria: unmarried, regular menstrual cycles (21-35 days, lasting 2-7 days), no psychiatric or medical disorders (e.g., thyroid disease, diabetes, PCOS)	PMDD: assessed using a predesigned proforma based on the DSM-V diagnostic checklist.	QoL: assessed using WHOQOL-BREF scale	- Prevalence: 12.1% of female medical students were diagnosed with PMDD.<br>- PMDD students reported significantly lower scores in physical and psychological health (p < 0.001).<br>- A significant proportion required medical treatment to function daily compared to healthy peers (p = 0.001).<br>- Work capacity and satisfaction were significantly lower in PMDD students.<br>- Fifth-year students had the	Confounders not adjusted in PMS-QoL association

© 2025 Wang Q et al. *JAMA Network Open*.

First author last name, year	**Study design**<br>**Data source**<br>**Study population**	Exposure	Outcome	Main findings	Notes
	Exclusion: Students with psychiatric disorders or medical conditions			highest proportion of PMDD cases (16.7%).	
Al-Shahrani, 2021	**Study Design:**<br>Cross-sectional study<br>**Data Source:**<br>Self-reported data<br>**Study Population:**<br>388 female students from Faculty of Medical Applied Sciences and Faculty of Medicine at Bisha University, Saudi Arabia, aged 18-25 years	PMS: diagnosed using the Premenstrual Syndrome Scale (PSS), based on the American Psychiatric Association criteria.	Health-related QoL: Health-related QoL questionnaire, assessing domains such as general well-being, study satisfaction, stress from university study conditions, and homework interface	- 64.9% of students were diagnosed with PMS.<br>- Significant PMS-related symptoms included depressive affect and anxiety, fatigue and irritability, and bloating or tender breasts.<br>- Menstruation significantly impacted QoL, particularly the homework interface	Confounders not adjusted in PMS-QoL association
Yamada, 2017	**Study Design:**<br>Retrospective design<br>**Data Source:**<br>Survey data from department of Psychiatry, Tokyo Women's Medical University, Medical Center East, Japan<br>**Study Population:**<br>66 untreated female patients with PMDD<br>Aged 18 years or older (mean age: 31.9 ± 7.2 years)	PMDD: Diagnosed based on DSM-IV-TR criteria<br>(Symptoms assessed during premenstrual and postmenstrual phases)	QoL: EuroQoL-5D (EQ-5D)	- 65% of patients exhibited a "V-type" symptom pattern (gradual onset and rapid resolution post-menses), while 35% had a "U-type" pattern (sustained low QoL until menstruation).<br>- PMDD results in a significant QALY reduction compared to the general population (0.933 vs. 0.795).<br>- The QALY loss in PMDD patients is comparable to other mental	Confounders not adjusted in PMS-QoL association

© 2025 Wang Q et al. *JAMA Network Open*.

First author last name, year	Study design<br>Data source<br>Study population	Exposure	Outcome	Main findings	Notes
				disorders like major depressive disorder and generalized anxiety disorder.	
Jaber, 2022	**Study Design:**<br>Cross-sectional study<br>**Data Source:**<br>Self-reported data<br>**Study Population:**<br>179 women aged 15–45 years from Family medicine walk-in clinic, University of Jordan Hospital, Amman, Jordan;<br>Exclusion: Pregnant women, those who gave birth in the last 6 months, or those on hormonal contraception	Premenstrual disorder(PMD): Arabic validated version of the shortened Premenstrual Assessment Form (SPAF)	QoL: a 7-item quality of life questionnaire that was developed by the researchers	- 88% of women experienced PMS, with most seeking help from relatives (51%), followed by physicians (34%)<br>- No significant association between PMS severity and seeking consultation (p = 0.194).<br>-PMS symptoms significantly affected daily activities, satisfaction with general appearance and weight, and relationships with family members and others	Confounders not adjusted in PMS-QoL association
Branecka-Woźniak, 2022	**Study design**: Cross-sectional study<br>**Data Source:** self-reported data from Conducted at St. Maksymilian Maria Kolbe Catholic Secondary School in Szczecin, Non-Public Healthcare Center "MEDI-PLUS" in Zwierzyno, and	PMS and PMDD:<br>PMS assessed using an author-developed questionnaire based on DSM-IV criteria for PMDD;<br>PMDD diagnosis required ≥11 out of 15 symptoms rated as	QoL: Measured using the standardized WHOQOL-BREF questionnaire.	- Prevalence: PMS: 40.7% (94 women); PMDD: 9.1% (21 women).<br>- Symptom Severity: Emotional symptoms were most severe (mean: 3.17, p = 0.010), followed by behavioral (3.00) and somatic (2.70) symptoms.<br>- Quality of Life: Overall QoL was	Confounder Adjustment: Not explicitly performed

© 2025 Wang Q et al. *JAMA Network Open*.

First author last name, year	Study design<br>Data source<br>Study population	Exposure	Outcome	Main findings	Notes
	through electronic means (July–November 2018).<br>**Study Population:** 231 regularly menstruating women aged 18 and older, with no prospective symptom tracking or exclusion of other conditions (e.g., depression, anxiety, thyroid disease).	significant or severe on a 5-point scale.		generally at a medium level. The lowest scores were in psychological health (mean: 11.61, p = 0.006), and the highest were in social relationships (mean: 13.76, p = 0.002).	
Uran, 2017	**- Study Design**: Cross-sectional study<br>**- Data Source**: self- reported data from Ankara University Child and Adolescent Psychiatry Department, Turkey (3 months duration).<br>**- Study Population:**<br>55 adolescent girls (13–18 years) admitted for non-PMS related symptoms. Exclusions included prior psychiatric/gynecologic admissions, chronic illness, birth control use, smoking, alcohol/drug use, and abnormal thyroid or	PMS: diagnosis and severity were determined using the Premenstrual Assessment Form (PAF), a retrospective self-report questionnaire.	HRQoL: Measured using the Pediatric Quality of Life Inventory (PedsQL) for adolescents and parent proxies.	- PMS Impact: Adolescents with PMS had significantly lower HRQoL scores than those without PMS (63.3 vs. 73.9, p = 0.03), with severe PMS showing the lowest scores (51.6, p = 0.01).<br>- Psychiatric Comorbidity: 89% had at least one psychiatric disorder, most commonly anxiety (38%) and major depressive disorder (27%).<br>- Correlations: HRQoL scores negatively correlated with PMS severity (r = –0.395 to –0.401, p < 0.01).	Confounder Adjustment: Not explicitly performed

© 2025 Wang Q et al. *JAMA Network Open*.

First author last name, year	Study design<br>Data source<br>Study population	Exposure	Outcome	Main findings	Notes
	hemoglobin levels.				
Delara, 2012	**- Study Design:** Cross-sectional study<br>**- Data Source:** self-reported data collected in Sabzevar boarding high schools, Khorasan province, Iran<br>**- Study population:** 602 female adolescents aged 14–19 years<br>Inclusion: Students from three boarding high schools<br>Exclusion: Students with irregular menstrual cycles, major medical/psychological issues, hormonal therapy, or recent catastrophes	PMS: A designed questionnaire based on the ICD-10 for PMS diagnosis (at least one distressing premenstrual symptom).<br><br>PMDD: A designed questionnaire based on Diagnostic and Statistical Manual, 4th edition (DSM-IV) for PMDD diagnosis	HRQoL: Measured using the SF-36	- All participants met the ICD-10 criteria for PMS, and 37.2% had PMDD.<br>- Students with PMDD showed significantly lower HRQoL scores, with the greatest differences in role emotional (43.65 vs. 69.71), role physical (52.27 vs. 74.35), bodily pain (58.11 vs. 73.37), and social functioning (61.30 vs. 77.62) (all $p < 0.001$).	Confounder Adjustment: Adjusted for age.
Yang, 2008	**Study Design**<br>Cross-sectional study.<br>**Data Source**<br>Data were collected through an online survey (Zoomerang.com) from a nationwide panel (ZoomPanel).<br>**Study Population**	PMDD: evaluated by Retrospective Diagnostic and Statistical Manual of Mental Disorders, 4th Edition, Text Revision (DSM-IV-TR) diagnostic criteria	HRQoL: measured using the SF-12v2 Health Survey, covering eight domains and two summary measures	- Women at risk for PMDD had significantly lower HRQoL scores, particularly in bodily pain (BP) and mental health (MH) domains, compared to general population norms.<br>- HRQoL scores for the PMDD cohort were significantly below age- and comorbidity-adjusted	The study used regression models to adjust for age and chronic diseases but did not report the

© 2025 Wang Q et al. *JAMA Network Open*.

First author last name, year	Study design<br>Data source<br>Study population	Exposure	Outcome	Main findings	Notes
	Women aged 18–45 from the U.S., with inclusion criteria such as fluent English, regular menstrual cycles, no recent use of antidepressants, and no professional treatment for mental health or substance use disorders in the last two years. Final sample size: 971 women			norms across six SF-12v2 scales (RF, BP, VT, SF, RE, MH) and both summary measures.<br>- PMDD burden on HRQoL was greater than back pain, similar to type 2 diabetes, hypertension, OA, and RA, and comparable to depression.<br>- The impact of PMDD was more pronounced in mental/emotional HRQoL than in physical HRQoL.	coefficients.
Balık, 2015	**- Study Design**<br>Cross-sectional study<br>**- Data Source**<br>Self-reported data collected from at the gynecology clinic of Recep Tayyip Erdogan University Medicine School, Turkey (May 2012 to March 2013).<br>- **Study Population**<br>Total: 89 women aged 17–45 years with regular menstrual cycles.<br>Exclusion: Psychotropic drug use, history of psychiatric	**-** PMS: Symptoms present for at least two days during the luteal phase indicate PMS.<br>**-** PMDD: Confirmed over two menstrual cycles using the Daily Record of Severity of Late Luteal Phase Problems scale.	Anxiety, depression, quality of life, and disability.<br>Evaluation Tools:<br>Hospital Anxiety and Depression Scale (HADS) for anxiety and depression.<br>SF-36 for QoL (physical and mental health components).<br>Brief Disability Questionnaire (BDQ) for physical and social disability.	- PMDD group had significantly higher HADS anxiety and depression scores than the PMS group.<br>- Both groups showed medium disability levels, but no significant differences in BDQ scores between the groups.<br>- PMDD group had significantly lower SF-36 scores across all domains compared to the PMS group (p<0.01).<br>- PMS and PMDD negatively affected quality of life, with PMDD causing greater impairment.	Confounder Adjustment: Not explicitly performed

© 2025 Wang Q et al. *JAMA Network Open*.

First author last name, year	Study design<br>Data source<br>Study population	Exposure	Outcome	Main findings	Notes
	illness in the last 6 months, menstrual irregularity, oral contraceptive or hormone therapy use, alcohol or drug abuse.				
Sahin, 2014	***Study Design:***<br>Cross-sectional study<br>**Data Source:**<br>Self-reported data from Sakarya University, Turkey, between October 25, 2012, and April 25, 2013.<br>**Study Population:**<br>Female university students<br>Sample size: 1008 students<br>Median age: 21 years	PMS: Evaluated using the Premenstrual Syndrome Scale (PSS) based on DSM-III and DSM-IV-R criteria.	QoL: assessed using the SF-36.	- The frequency of PMS was 36.3%.<br>- PMS was associated with significantly lower scores in all QoL domains ($p<0.05$).	Confounders not adjusted in PMS-QoL association
Ogawa, 2024	**Study Design:**<br>Cross-sectional study<br>**Data Source:**<br>Conducted at the Department of Obstetrics and Gynaecology, Tokyo Dental College Ichikawa General Hospital, Japan<br>**Study Population**<br>55 Women seeking treatment	PMD and PME:<br>The diagnosis of core PMD and PME was based on the ISPMD consensus and the Green Top Guidelines using the Daily Record of Severity of Problems	Mental health: assessed using the Hospital Anxiety and Depression Scale (HADS)<br>Anxiety and depression subscales (scores range from 0 to 21)<br>QoL: assessed using the SF-36 Health	- About one-third of the patients diagnosed with PMDs were found to have PME<br>- the PME group had lower scores than the core PMD group in all domains of QoL except physical and social functioning.	Confounders not adjusted in PMS-QoL association

© 2025 Wang Q et al. *JAMA Network Open*.

First author last name, year	Study design<br>Data source<br>Study population	Exposure	Outcome	Main findings	Notes
	for premenstrual symptoms		Survey		
Mushtaq, 2020	**Study Design:**<br>Cross-sectional study<br>**Data Source:**<br>Survey data from Rawalakot, Azad Kashmir, Pakistan<br>**Study Population:**<br>Married women aged 20-45 years<br>*Sample size:* 300 participants<br>*Inclusion criteria*: Minimum education level of 8th grade, intact family structure<br>*Exclusion criteria:* Divorce, separation, psychopathology, serious medical conditions, and irregular menstrual cycles	PMS: Evaluated using the Urdu version of Greene Climacteric Symptoms Scale (GCSS)	QoL: Measured using the Urdu version of the World Health Organization Quality of Life (WHOQOL-BREF) scale	- Premenstrual symptoms were negatively correlated with all QoL domains<br>- Somatic symptoms were the most significant negative predictor of QoL:	Confounder Adjustment: Not performed
Lustyk, 2004	**Study Design:**<br>Cross-sectional study<br>**Data Source:**<br>Self-reported data collected from Seattle Pacific University, Seattle, WA, USA<br>Participants were recruited from an introductory	PMS: Evaluated using the SPAF	Stress assessed using the Student Stress Questionnaire<br>QoL measured using the Quality of Life Inventory (QOLI)<br>Physical Activity measured using the	- Women with high PMS had significantly more stress (mean score 326 vs. 244, p=0.002) and poorer QoL compared to those with low PMS.<br>- Significant differences were found in QoL subdomains: Lower self-esteem (p=0.03); Reduced leisure	Confounder Adjustment: Not performed

© 2025 Wang Q et al. *JAMA Network Open*.

**First author last name, year**	**Study design**<br>**Data source**<br>**Study population**	**Exposure**	**Outcome**	**Main findings**	**Notes**
	psychology class<br>**Study Population:**<br>114 Female college students aged 18-33 years		Godin Leisure Time Activity Questionnaire,	time activity (p=0.05)<br>- No significant differences between groups in physical activity levels (frequency, volume, intensity)	

© 2025 Wang Q et al. *JAMA Network Open*.

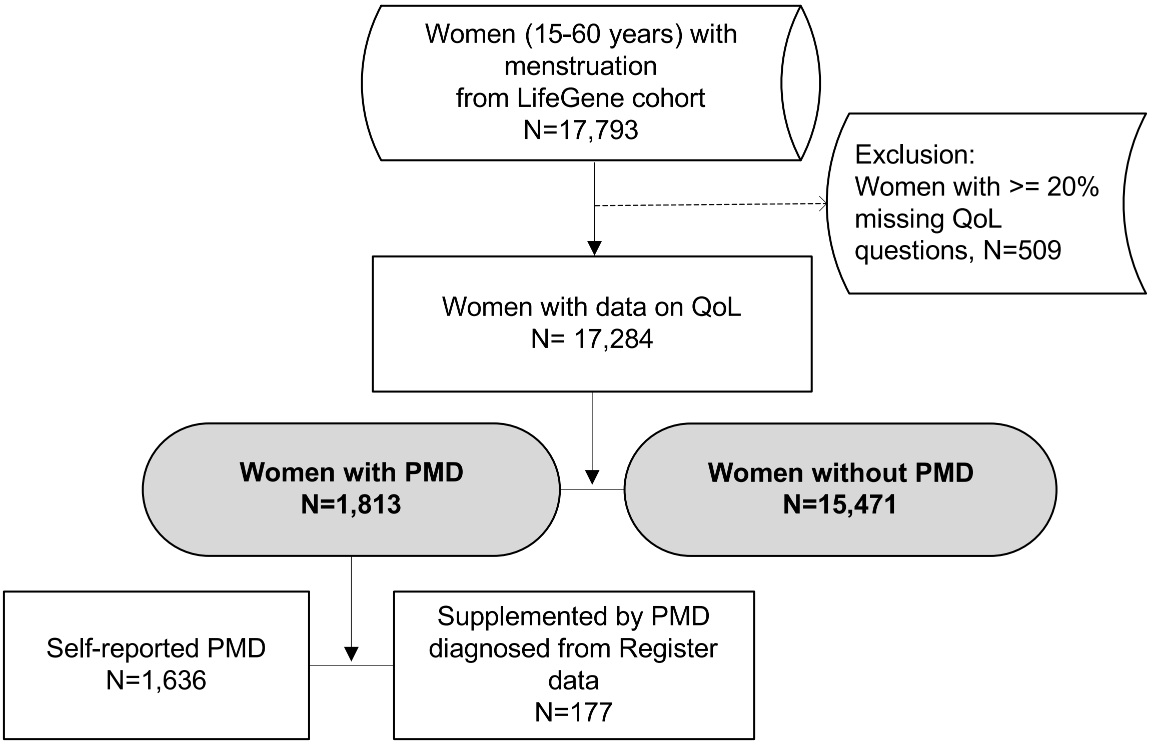

Figure 1 Flow chart over study population

Abbreviations: PMD, premenstrual disorder; QoL, quality of life

© 2025 Wang Q et al. *JAMA Network Open*.

eTable 2 Codes for identification of premenstrual disorders, psychiatric and somatic comorbidities

Diagnosis	Source	Type	Codes
Premenstrual disorders	NPR, SPCR	ICD-10	N943, 625E
	PDR	ATC	N06AB, N06AX, N06AA, G03A, G02B Associated with a Swedish written indication for PMDs: "PMS", "PREMENSTRUELLT SYNDROM", "PREMENSTRUELLT DYSFORSIKT SYNDROM", "PREMENSTRUELLT DYSFORI", "PMD", "PMDD", "PMDS", "MENS"
Psychiatric comorbidity	NPR, SPCR	ICD-10	F10-F99 excluding PMD
Charlson Index	NPR	ICD-8,9,10	Adaptation of the Charlson Comorbidity Index for Register-Based Research in Sweden[1]

Abbreviations: NPR, National Patient Register; SPCR: Stockholm Primary care register; ICD, International Classification of Diseases; PDR, National Prescribed Drug Register; ATC: Anatomical Therapeutic Chemical; PMDs, Premenstrual Disorders.

[1] ICD codes provided by Ludvigsson JF, Appelros P, Askling J, Byberg L, Carrero JJ, Ekström AM, et al. Adaptation of the charlson comorbidity index for register-based research in sweden. Clin Epidemiol. 2021;13:21-41

eMethods

Screening Criteria in Premenstrual Symptom Screening Tool(PSST) for Assessing PMDs

The questionnaire was modified to begin with three screening questions: *1) "During most menstrual cycles in the past year, have you experienced mood changes and/or physical symptoms during the week before menstruation?"; 2) "Have your premenstrual symptoms been so severe that they have interfered with your relationships with others or your ability to perform work or other activities?"*. If participants answered 'yes' to both questions, they were then asked 3*) "Are you absolutely sure that the symptoms are limited to the premenstrual period, meaning that you're always completely symptom-free about a week after your period starts?"*.

Classification of PMD subtypes in PSST

Participants were classified as having severe PMS if they had: (1) ⩾1 out of 4 affective symptoms rated as moderate to severe; (2) ⩾ 4 other symptoms rated as moderate to severe; and (3) ⩾1 symptom moderately to severely impacting relationships or social activities. Participants were classified as having PMDD if they had: (1) ⩾1 out of 4 affective symptoms rated as severe; (2) ⩾4 other symptoms rated as moderate to severe; and (3) ⩾1 symptom severely impacting relationships or social activities.

Definition and Classification of Childhood Abuse and Stressful Life Events

Based on a modified Life Stressor Checklist-Revised (LSC-R) questionnaire (see below), childhood abuse was defined as experiencing any of the following events before age 18, including witnessing family abuse, being a victim of non-sexual or sexual assault, and being coerced into sexual activity. Stressful life events in adulthood were assessed across the lifespan, including financial difficulties, discrimination, bullying, accidents, natural disasters, imprisonment, bereavement, serious illness, emotional abuse, separation from a child, experiences of violence, harassment, and forced sexual activity. Exposure levels were categorized by the number of events experienced.

Modified Life Stressor Checklist – Revised:

1. Have any of the following happened to you during your lifetime? (Mark all that apply)

☐ Your own divorce or separation
☐ Serious financial problems (e.g., no money for food)
☐ Experienced discrimination in a very distressing way
☐ Been subjected to bullying
☐ Witnessed a serious accident
☐ Been involved in a serious accident
☐ Experienced a natural disaster (e.g., tsunami, hurricane, forest fire)
☐ Been imprisoned for a crime
☐ A close family member has been imprisoned
○ None of the above
○ Don't know / Prefer not to answer

2. Have any of the following happened to you during your lifetime? (Mark all that apply)

☐ A close friend has died
☐ Been adopted or placed in a foster home

© 2025 Wang Q et al. *JAMA Network Open.*

☐ *Serious physical or mental illness*
☐ *A close family member with a serious physical or mental illness*
☐ *A close family member has died (not a child)*
☐ *A child has died*
☐ *Separated from your child against your will*
☐ *Before age 18, witnessed abuse between family members*
☐ *Physically neglected (e.g., not given food)*
☐ *Emotionally abused or neglected (e.g., often made to feel ashamed)*
○ *None of the above*
○ *Don't know / Prefer not to answer*

3. Have you experienced any of the following? (Mark all that apply)
☐ *Witnessed a robbery or mugging*
☐ *Been a victim of a robbery or mugging*
☐ *Been stalked, threatened with death, or seriously injured*
☐ *Before age 18, have been physically abused or mistreated (not sexually) by someone you knew*
☐ *As an adult, have been physically abused or mistreated (not sexually) by someone you knew*
☐ *Been subjected to or harassed with sexual advances*
☐ *Before age 18, have been subjected to sexual abuse or forced into sexual acts against your will*
☐ *As an adult, have been subjected to sexual abuse or forced into sexual acts*
☐ *Forced to have sex before age 18*
☐ *Forced to have sex as an adult*
☐ *Had sex in exchange for money or drugs*
○ *None of the above*
○ *Don't know / Prefer not to answer*

eTable 3 Comparison of characteristics between included and excluded women

Characteristic	Women, N (%)	
	Included N=17,284	Excluded N=509
PMD		
Yes	1,813 (10.5)	25 (4.9)
No	15,471 (89.5)	484 (95.1)
Age at survey, year		
Mean(SD)	32.4 (8.5)	30.0 (9.4)
15-24	3,359 (19.4)	154 (30.3)
25-34	7,488 (43.3)	200 (39.3)
35-44	4,555 (26.4)	112 (22.0)
45-54	1,821 (10.5)	41 (8.1)
55-60	61 (0.4)	2 (0.4)
BMI(kg/m²)		
<18.5	552 (3.2)	9 (1.8)
18.5-25	12,114 (70.1)	142 (27.9)
25-29	2,581 (14.9)	33 (6.5)
>=30	519 (3.0)	4 (0.8)
unknown	1,518 (8.8)	321 (63.1)
Country of birth		
Sweden	15,435 (89.3)	448 (88.0)
Other	1,849 (10.7)	61 (12.0)
Region of residence		
Stockholm	13,285 (76.9)	382 (75.1)
other	3,999 (23.1)	127 (25.0)
Civil status		
Partnered	6,619 (38.3)	225 (44.2)
Single	10,660 (61.7)	271 (53.2)
Unknown	5 (0.0)	13 (2.6)
Education level		
Pre-secondary	156 (0.9)	66 (13.0)
Secondary	3,333 (19.3)	106 (20.8)
Post-secondary	12,258 (70.9)	290 (57.0)
Postgraduate	1,531 (8.9)	17 (3.3)
Unknown	6 (0.0)	30 (5.9)

© 2025 Wang Q et al. *JAMA Network Open*.

Income		
Q1	4,312 (25.0)	127 (25.0)
Q2-Q3	8,627 (49.9)	248 (48.7)
Q4	4,308 (24.9)	121 (23.8)
Unknown	37 (0.2)	13 (2.6)
Alcohol drinking		
Never	569 (3.3)	15 (3.0)
Monthly	9,029 (52.2)	230 (45.2)
Weekly	7,248 (41.9)	126 (24.8)
Unknown	438 (2.5)	138 (27.1)
Smoking status		
Never	5,838 (33.8)	151 (29.7)
Former smokers	9,843 (57.0)	228 (44.8)
Current smokers	1,417 (8.2)	39 (7.7)
Unknown	186 (1.1)	91 (17.9)
Childhood abuse		
Yes	2,983 (17.3)	30 (5.9)
No	13,849 (80.1)	240 (47.2)
Unknown	452 (2.6)	239 (47.0)
Stressful life events		
0 events	3,323 (19.2)	111 (21.8)
1 event	4,273 (24.7)	50 (9.8)
2-3 events	5,789 (33.5)	71 (14.0)
>3 events	3,433 (19.9)	38 (7.5)
Unknown	466 (2.7)	239 (47.0)
Parity		
0	11,221 (64.9)	338 (66.4)
1-2	4,968 (28.7)	141 (27.7)
>=3	1,095 (6.3)	30 (5.9)

Abbreviations: PMD: premenstrual disorder; BMI: body mass index; N, number of events; SD, standard deviation.

© 2025 Wang Q et al. *JAMA Network Open*.

eTable 4 Association between PMDs and quality of life, assessed by visual analog scale (VAS) score

	No PMDs N=15,471	PMDs N=1,813	Model 1[a]	Model 2[b]	Model 3[c]
	Mean (SD)	Mean (SD)	Difference in mean score(95%CI)		
VAS score [d]	0.02 (1.00)	-0.16 (1.00)	-0.18 * (-0.23, -0.13)	-0.17 * (-0.22, -0.12)	-0.11 * (-0.16, -0.07)

Abbreviations: PMD, premenstrual disorder; SD, standard deviation; CI, confidence interval; VAS, visual analog scale.

[a] Model 1 was a crude model

[b] Model 2 was adjusted for age, country of birth, region of residence, civil status, educational level, and income.

[c] Model 3 was further adjusted for body mass index, alcohol drinking, smoking status, childhood abuse, stressful life events, and parity.

[d] VAS score represented self-assessed health status on a scale of 0 to 10, with a higher score indicating better health. The scores were standardized into z-scores.

* $P<0.05$

© 2025 Wang Q et al. *JAMA Network Open*.

eTable 5 Association between PMDs and quality of life, restricted to PMDs ascertained in both clinical diagnoses and questionnaire assessment

	No PMDs N=15,471	PMDs N=420	Model 1[a]	Model 2[b]	Model 3[c]
	Mean (SD)	Mean (SD)	Difference in mean score(95%CI)		
Total score [d]	-0.03 (0.99)	0.30 (1.00)	0.33 * (0.23, 0.42)	0.31 * (0.21, 0.40)	0.25 * (0.16, 0.35)
Total score without anxiety/depression item	-0.02 (0.99)	0.13 (0.98)	0.15 * (0.05, 0.24)	0.12 * (0.02, 0.21)	0.07 (-0.02, 0.17)
Total score without pain/discomfort item	-0.03 (0.99)	0.30 (0.96)	0.33 * (0.23, 0.42)	0.32 * (0.23, 0.42)	0.27* (0.18, 0.37)

Abbreviations: PMD, premenstrual disorder; SD, standard deviation; CI, confidence interval

[a] Model 1 was a crude model

[b] Model 2 was adjusted for age, country of birth, county of residence, civil status, educational level, and income.

[c] Model 3 was further adjusted for body mass index, alcohol drinking, smoking status, childhood abuse, stressful life events, and parity.

[d] The total score was calculated by summing responses to all five questions in the EQ-5D scale, with a higher score indicating poorer quality of life, and then converted to z-score.

* P<0.05

© 2025 Wang Q et al. *JAMA Network Open*.

eTable 6 Association between PMDs and quality of life, a complete case analysis[a]

	No PMDs N=15,286	PMDs N=1,793	Model 1[b]	Model 2[c]	Model 3[d]
	Mean (SD)	Mean (SD)	Difference in mean score(95%CI)		
Total score[e]	-0.03 (0.99)	0.27 (1.03)	0.30 * (0.26, 0.35)	0.28 * (0.24, 0.34)	0.22 * (0.17, 0.27)
Total score without anxiety/depression item	-0.02 (0.99)	0.16 (1.06)	0.18 * (0.13, 0.23)	0.15 * (0.11, 0.20)	0.10 * (0.05, 0.15)
Total score without pain/discomfort item	-0.03 (0.99)	0.25 (1.04)	0.28 * (0.23, 0.33)	0.28 * (0.23, 0.33)	0.22 * (0.17, 0.27)
EQ value [f]	0.92 (0.06)	0.91 (0.07)	-0.02 * (-0.02, -0.01)	-0.02 * (-0.02, -0.01)	-0.01* (-0.02, -0.01)
EQ value (z-score)	0.03 (0.99)	-0.23 (1.08)	-0.26 * (-0.31, -0.21)	-0.25 * (-0.30, -0.20)	-0.18 * (-0.23, -0.13)

Abbreviations: PMD, premenstrual disorder; SD, standard deviation; CI, confidence interval.

[a] In the complete case analysis, we excluded 205 individuals with any missing values in the assessment of quality of life. Among the analyzed participants, 15,286 (89.5%) were classified as non-PMD, while 1,793 (10.5%) were deemed as PMD.

[b] Model 1 was a crude model

[c] Model 2 was adjusted for age, country of birth, county of residence, civil status, educational level, and income.

[d] Model 3 was further adjusted for body mass index, alcohol drinking, smoking status, childhood abuse, stressful life events, and parity.

[e] The total score was calculated by summing responses to all five questions in the EQ-5D scale, with a higher score indicating poorer quality of life, and then converted to z-score.

[f] EQ value was based on the Swedish value set: https://pubmed.ncbi.nlm.nih.gov/23975375/

* P<0.05

© 2025 Wang Q et al. *JAMA Network Open*.

eTable 7 Sensitivity analysis: Association between PMDs and quality of life stratified by comorbidities[a]

		Total score without anxiety/depression item		Total score without pain/discomfort item	
		Mean, SD	Difference in mean score (95% CI)[a]	Mean, SD	Difference in mean score (95% CI)[a]
Psychiatric comorbidities					
No	No PMDs	-0.10 (0.90)	Ref.	-0.17 (0.86)	Ref.
	PMDs	0.04 (0.96)	0.07 (0.02, 0.13) *	0.06 (0.91)	0.19 (0.13, 0.24) *
Yes	No PMDs	0.27 (1.22)	0.27 (0.23, 0.30) *	0.48 (1.23)	0.56 (0.52,0.60) *
	PMDs	0.40 (1.26)	0.35 (0.27, 0.43) *	0.62 (1.17)	0.68 (0.60,0.75) *
P for interaction[b]			*0.89*		*0.15*
Somatic comorbidities					
No	No PMDs	-0.06 (0.94)	Ref.	-0.05 (0.96)	Ref.
	PMDs	0.09 (0.98)	0.07 (0.01, 0.12) *	0.21 (0.99)	0.20 (0.15, 0.25) *
Yes	No PMDs	0.30 (1.26)	0.28 (0.23, 0.33) *	0.17 (1.19)	0.16 (0.11, 0.21) *
	PMDs	0.60 (1.46)	0.52 (0.40, 0.64) *	0.45 (1.25)	0.39 (0.27, 0.51) *
P for interaction			*0.01*		*0.57*

Abbreviations: SD, standard deviation; CI, confidence interval; PMD, premenstrual disorder.

[a] Models were adjusted for age, country of birth, region of residence, civil status, educational level, and income, body mass index, alcohol drinking, smoking status, childhood abuse, stressful life events, and parity.

[b] P-values for interaction were obtained from F-tests of linear hypotheses in the Ordinary Least Squares regression model.

* P<0.05

© 2025 Wang Q et al. *JAMA Network Open*.
