# Supplementary material for: Premenstrual Disorders and Quality of Life in Sweden
Source: JAMA Netw Open. 2025 Sep 23;8(9):e2533823. doi: 10.1001/jamanetworkopen.2025.33823 (PMC12457971; doi:10.1001/jamanetworkopen.2025.33823)
Supplement: Supplement 2. — Data Sharing Statement [file jamanetwopen-e2533823-s002.pdf]

Data Sharing Statement

Due to privacy protection measures, such as the General Data Protection Regulation (GDPR), the cohort's information is not publicly accessible. Access to LifeGene resources is granted only after an ethical evaluation by the relevant authorities (https://ki.se/en/research/research-infrastructure-and-environments/core-facilities-for-research/ki-biobank-core-facility-kibb/lifegene, email: sara.hagg@ki.se). For further information on acquiring access to Swedish register data, consult the Swedish National Board of Health and Welfare's website (https://bestalladata.socialstyrelsen.se/, email: registerservice@socialstyrelsen.se) and/or the Statistics Sweden website (https://www.scb.se/vara-tjanster/bestall-data-och-statistik/, email: scb@scb.se).
Data available: No
